# Investigation of reflectance, transmittance, and thermal response of skin and surface-textured skin-equivalent phantoms in the terahertz frequency range around 0.3 THz

**DOI:** 10.1038/s41598-025-28863-0

**Published:** 2025-12-22

**Authors:** Shota Yamazaki, Masafumi Fukunari, Yoshinori Tatematsu, Yujiro Kushiyama, Tomoaki Nagaoka, Maya Mizuno

**Affiliations:** 1 National Institute of Information and Communications Technology, Koganei, Tokyo, 184–8795 Japan; 2 Research Center for Development of Far-Infrared Region, University of Fukui, Bunkyo, Fukui, 910–8507 Japan

**Keywords:** Terahertz, Skin, Skin-equivalent phantom, Reflectance, Transmittance, Thermal response, Materials science, Optics and photonics, Physics

## Abstract

In this study, we investigated the electromagnetic and thermal responses of a skin-equivalent phantom with surface roughness under terahertz (THz)-wave exposure. Reflectance and transmittance were measured and compared with those of porcine skin, surface roughness standards, and flat phantoms. Results showed that both reflectance and transmittance were reduced owing to surface roughness on dielectrics. To evaluate the thermal effects of THz absorption in a skin phantom, we fabricated phantoms incorporating temperature-sensitive fluorescent probes and irradiated them with 265 GHz waves at power densities of 100, 150, and 200 mW/cm². High-resolution temperature mapping using confocal laser scanning microscopy revealed that temperature elevation occurred up to a depth of approximately 1 mm. Moreover, phantoms with surface structures showed consistently higher temperature increases than flat phantoms. These findings imply that the dominant factors affecting THz–tissue interactions vary with exposure duration. Under short-term irradiation, effective optical properties such as reflectance and transmittance altered by roughness may play a more significant role. In contrast, during long-term exposure, reduced surface water content increases transmitted THz power density into deeper tissue regions, enhancing energy absorption below the surface. Careful consideration of these factors can improve the accuracy of phantom-based evaluations in THz exposure assessments.

**Supplementary Information:**

The online version contains supplementary material available at 10.1038/s41598-025-28863-0.

## Introduction

The development of terahertz (THz) technologies has been accelerated for radio communications^1,2^. For example, recent work has demonstrated rapid inverse design of high Q-factor THz filters, highlighting the increasing relevance of THz devices in future wireless systems^3,4^. Toward the use of THz technologies in future-generation wireless communication, it is also necessary to investigate the possible health effects of THz waves on the human body. Although the THz effects on biomatter can be split into two broad categories, namely, thermal and nonthermal effects^5^, the reproducibility of nonthermal effect research data is poor, and only the thermal effects (temperature increase) are considered in the international commission guidelines^6,7^. In addition, research data on thermal effects at THz frequencies is limited compared with that at other frequencies, where practical use is in progress. The accumulation of thermal effect research data obtained by biological investigations and physical analyses of exposure properties is therefore required. Thus, we have been conducting analyses of the exposure properties of biological tissues exposed to THz waves by simulations using tissue models^8,9^ and are planning experiments using biological-tissue-equivalent phantoms for the eyes and skin, where most THz waves are absorbed. In experiments using biological-tissue-equivalent phantoms, a simple flat design is frequently applied in the radio frequency region, owing to the long wavelength. However, surface roughness such as skin bumps and wrinkles may affect the propagation properties of THz waves because their wavelengths are more similar to the skin roughness than those of other RF waves. In cases of wallpaper and concrete plaster with surface roughness, scattering effects were observed^10^. Therefore, the effect of skin roughness is expected to be investigated also in biological-tissue-equivalent phantoms. Furthermore, transmittance is particularly related to the temperature increase in skin caused by exposure to THz waves. The transmittance dependence on skin roughness will be helpful in discussing the exposure limits in guidelines.

In this study, by applying some THz technologies for nondestructive tests^11,12^ and techniques for examining skin tissues^13^, we experimentally investigated the THz reflectance and transmittance of skin-equivalent phantoms and compared them with those of other samples so that we can determine how surface roughness causes variations in optical properties. In addition to optical characterization, the experimental evaluation of temperature increase in skin-equivalent phantoms is also essential for understanding the thermal effects of THz wave exposure. In particular, since THz waves are strongly absorbed by water in biological tissues, even slight changes in surface structure or water content may alter energy deposition profiles and thermal responses in the skin. In this study, we fabricated a skin-equivalent phantom that reproduces the surface microstructure of human skin using a silicone mold and embedded it with temperature-sensitive fluorescent probes to visualize temperature distribution. By exposing the phantom to 265 GHz waves at controlled power densities, we were able to assess the impact of surface roughness on temperature elevation with high spatial resolution. This approach enables the direct investigation of how skin surface geometry affects both THz absorption and the resulting temperature increase, providing valuable insights for future thermal safety evaluations.

## Methods

### Measurement samples

For fundamental reflectance measurements, we used porcine skin tissues without fur (K1420, DARD. Co., Ltd.) and roughness standard pieces. The water contents of thirteen skin samples, which were measured using a moisture meter (Corneometer CM825, Courage + Khazaka), ranged from 24 to 46%. To investigate the reflectance and predict the transmittance of skin, skin-equivalent phantoms and three types of sandpaper composed of aluminum oxide, cotton, and polyester were used. The skin-equivalent phantoms were made with pure water, oil, glycerin fatty acid ester, and agar, which were mixed at mass ratios of 58, 30, 10, and 2%, respectively, to have a dielectric constant similar to that of skin. They were heated to approximately 100 °C and then mixed using a stirrer, followed by solidification at room temperature for approximately 60 min^14^ before use.

### Measurement system

Reflectance spectra of the samples were acquired using an attenuated-total-reflection-type THz time-domain spectroscopy (ATR-type THz-TDS) system (TAS7500TS, ADVANTEST Co.) or a reflection-type THz-TDS system (T-Ray 4000, Picometrix LLC (currently Luna Innovations Inc.)) at frequencies from 0.2 THz to 0.4 THz^14^. Both systems were commercially available and used without modifications. Detailed experimental configurations and procedures for these systems have been reported previously by Mizuno et al.^14^, who employed the same setup, ensuring that others can reproduce the measurements reliably. In the ATR-type TDS measurements of the skin, reflectance was calculated from the measured complex refractive index using the Fresnel formula of reflection. Sample surface is made to be smooth because sample is appressed to the ATR prism. The reflectance measured by the ATR-type THz-TDS system will therefore be called the reflectance of a smooth plane in this paper. For reference, the reflectance at normal incidence was also calculated using Fresnel’s equations with the complex refractive index *ñ *= *n* + *iκ*:

$$\:R=\mid\:( {\tilde{n}} _{1}\mathbf{}- {\tilde{n}}_{2}\mathbf{})/( {\tilde{n}} _{1}\mathbf{}+ {\tilde{n}} _{2}\mathbf{}){\mid\:}^{2}$$

For instance, with n_1_ = 1.0 + iκ_1_ with κ_1_ = 0 (air) and n_2_ = 2.1 + iκ_2_ with κ_2_ = 0.1–0.5 (epidermis-equivalent phantom^14^, the calculated reflectance is approximately 12–13%, which is in reasonable agreement with our measurements. In the reflection-type TDS measurements, time waveforms of THz pulses were recorded, and then the time waveforms were Fourier transformed to frequency domain intensities. Reflectance was calculated using the intensities of waves reflected from a sample or a metal plate at each frequency.

Optical configurations for the reflectance and transmittance measurements of samples with surface roughness are shown in Supplementary Fig. S1. For the reflection-type TDS (Supplementary Fig. S1, left), a polyethylene lens of 1-inch focal length and 35 mm diameter was used to vertically focus a THz beam on the sample surface, and the Rayleigh length of the THz beam was approximately 1.5 mm. When using the X–Y stage, the reflection-type TDS system can output a reflection image. Here, a pixel size was set to 0.5 mm. Reflection measurements were performed at room temperature. In transmittance measurements (Supplementary Fig. S1, right), a 280 GHz IMPATT source (IMPATT-280, Terasense Group Inc.) and a power meter (Erickson PM5, Virginia Diodes, Inc.) were used for THz generation and detection. THz waves were radiated from a diagonal horn to the free space and focused by parabolic mirrors. At the focal position, the waves were detected by an open-ended rectangular waveguide probe. The roughness of the samples used was confirmed using an optical coherence tomography (OCT) system (TEL210C1/M, Thorlabs). From the surface data obtained by OCT, the root mean square of roughness was calculated. Here, the mean roughness height was set to zero.

To measure the local power density set in exposure experiments using a gyrotron^15^, the same power meter was used as reported previously^16, together with an open-ended rectangular waveguide probe (AOEWGP-012E/3", Elmika).^

### Calculation method

In the calculation of the superposition of adjacent waves reflected from a sample with surface roughness, random roughness was assumed. Generally, the characteristic function φ of X (variable) complying with the Gaussian distribution of the standard deviation σ is expressed as

1 $$\:{\phi\:\left(t\right)=E[e}^{\left(itX\right)}]={e}^{\left(it\mu\:\right)-{\left(t\sigma\:\right)}^{2}/2},$$

where E is the expected value and µ is the average value. From Eq. (1), the superposition of the electric field of THz waves reflected from a rough plane (i.e., made a round trip) was statistically calculated as.

　

2 $$\:{E[e}^{\left(ikz\right)}]={e}^{-{\left(2k\sigma\:\right)}^{2}/2}$$

The average height < z > is zero, and k is the angular wavenumber. Because power is proportional to the squared electric field, the reflectance R from a random rough surface was derived by calculating

3 $$\:R={{R}_{s}e}^{-{\left(2k\sigma\:\right)}^{2}}$$

in this study. As the specular reflectance R_s_, the reflectance from metal or a slightly rough plane having a surface roughness of less than λ/32 was used (λ is the wavelength). The above equation can be converted to

4 $$\:R={R}_{s}{e}^{-{4\left\{\frac{k\left(2\sigma\:\right)}{2}\right\}}^{2}}.$$

Then, a part of curly brackets is called the Rayleigh roughness parameter^17^.

### Preparation of the phantoms for temperature measurement

Temperature sensing within the phantom was achieved using a ratiometric fluorescent polymeric thermometer (Funakoshi Co.)^18, incorporated into a transparent, skin-equivalent phantom with a modified composition (69.5% saline solution, 30% ethanol, and 0.5% agar) to enable fluorescence measurements. The fluorescent probe was mixed uniformly before gelation to ensure homogeneous distribution throughout the phantom.^

### Temperature measurements of phantoms

Phantoms containing the fluorescent thermoprobe were examined using a confocal microscope (FV3000, Olympus) equipped with a PLAPON1.25× objective (N.A. 0.04). Excitation was performed at 488 nm, and emission signals were collected at two spectral windows (490 nm and 620 nm), followed by image analysis with the cellSens software (cellSens Dimention Desktop 4.2.1, Olympus). The probe consists of a thermoresponsive polymer incorporating two fluorescent units: one whose intensity varies with temperature and another serving as a reference. Upon heating, conformational changes in the polymer alter the hydration state of the environment-sensitive fluorophore, leading to increased emission, whereas the reference fluorophore remains largely unaffected. This dual-emission design allows for ratiometric imaging of local temperature changes with high sensitivity, as reported previously^19^.

## Results

### Reflectance properties of Porcine skin

**Fig. 1:**
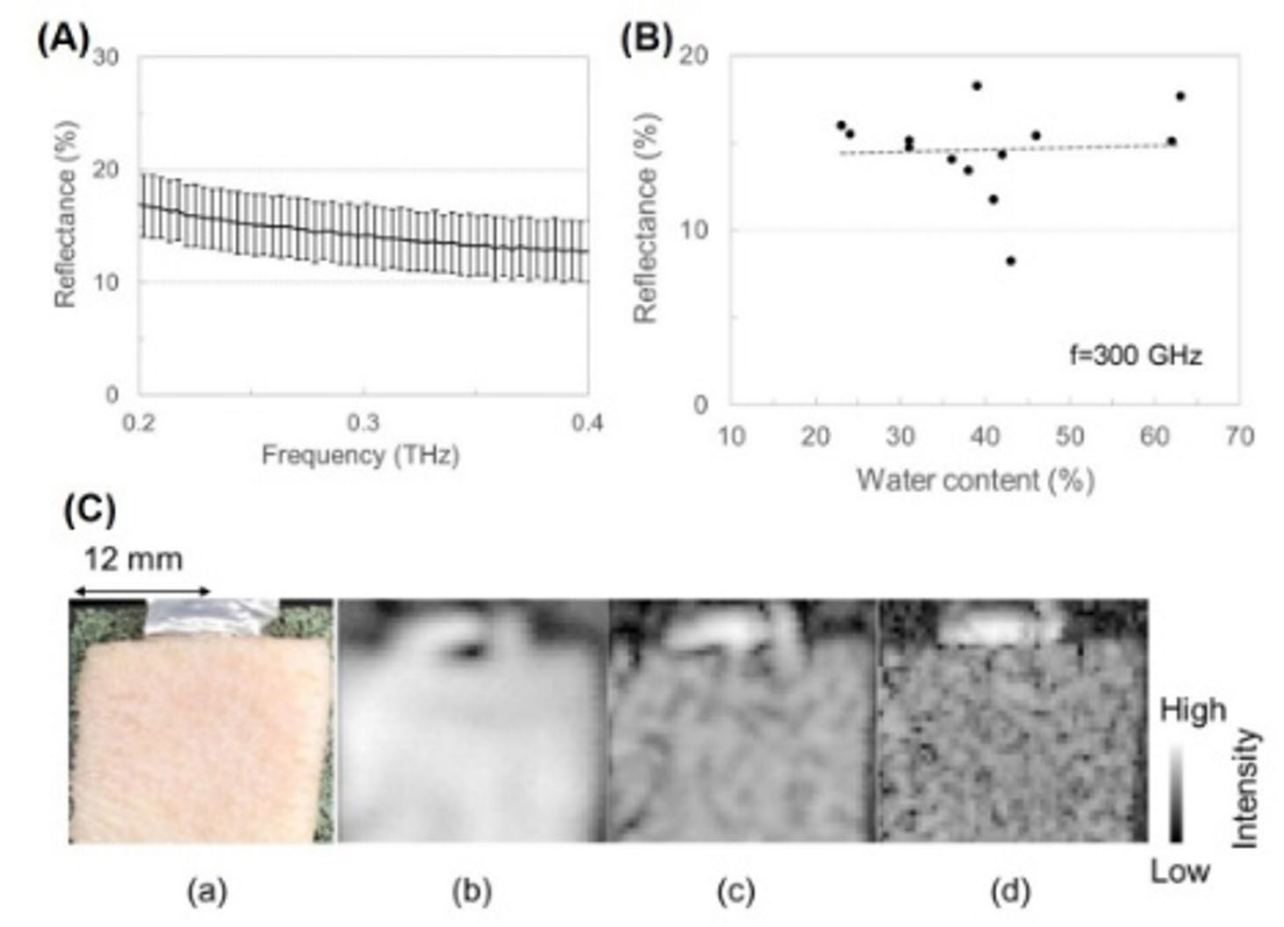
(**A**) Reflectance spectrum of porcine skin with water contents of 24–46%. (**B**) Relationship between reflectance and water content at 300 GHz. Error bars show the standard deviation (1σ) of ten measurements in the left graph. (**C**) Visible light image (a) and THz reflection images of porcine skin. THz reflection images were obtained in frequency bands of (b) 0.09–0.11 THz, (c) 0.19–0.21 THz, and (d) 0.29–0.31 THz.

First, the reflectance of the porcine skin with water contents from 24 to 46% was analyzed using the ATR-type THz-TDS system at a room temperature of approximately 24 °C (Fig. 1A). Thus, the refractive index of the skin with a smooth plane was measured, and then reflectance was calculated. Error bars indicate the standard deviations (1σ) of ten samples. The reflectance was approximately 14% at around 300 GHz. The standard deviations were less than ± 5% from 0.2 to 0.4 THz. The observed variability mainly reflects sample-to-sample differences in porcine skin, such as slight variations in surface shape and local hydration, which make it difficult to achieve a perfectly flat surface in ATR-THz measurements. This observation is consistent with previous reports on THz measurements of biological tissues^13,14^. Although the reflectance generally depends on water content, the reflectance values were almost the same within a deviation at 300 GHz, as shown in Fig. 1B. We therefore considered that the reflectance data at frequencies below 300 GHz could be separately discussed between surface roughness effects and a few individual deviations caused by water content.

To verify the reflectance distribution depending on the surface shape in the porcine skin, we acquired a THz reflection image of the skin with a water content of approximately 18% (dry tendency). Reflection intensities (squared electric field intensities) were integrated in a frequency band of 0.09–0.11 THz, 0.190–0.21 THz, or 0.29–0.31 THz at each pixel. In Fig. 1C, when the reflection intensity is high, the color is almost white. The image in the 0.1 THz band seems to show a no roughness, whereas that in the 0.3 THz band shows a wrinkle-like pattern. Furthermore, the reflection intensity decreases with increasing frequency to a greater extent than that shown in Fig. 1A. As is well known, the wavelength at 0.3 THz is approximately 1 mm, and the surface roughness caused by wrinkles is several tens of micrometers^20^ or more under a dry condition mentioned below. Thus, the space resolution for an imaging application is high, whereas the surface roughness gets closer to λ/8; the surface roughness could affect reflectance at frequencies over 0.3 THz.

**Fig. 2:**
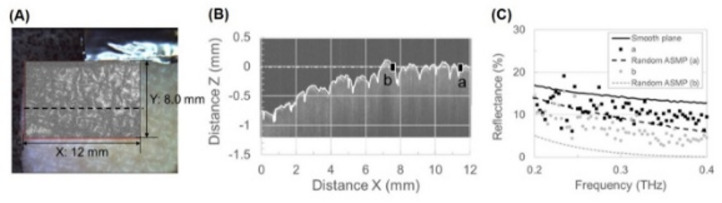
(**A**) OCT c-scan image overlapped with a visible light image. (**B**) OCT b-scan image of porcine skin. (**C**) Reflectance spectra of skin at *a* and *b* positions. Reflectance spectra measured by ATR-type TDS system (smooth plane) and calculated with assumption of random roughness.

From the above findings, we analyzed the correlation between the reflectance and surface roughness of the skin. Figure 2A shows an OCT c-scan image of the skin overlapped with a visible light image, and Fig. 2B shows an OCT b-scan image of the skin. The skin had a surface roughness [root mean square (RMS)] of approximately 0.03–0.15 mm. The roughnesses at the *a* (x = 9–12 mm) and *b* (x = 6–9 mm) positions were approximately 0.065 and 0.118 mm, respectively. At each position, a reflectance spectrum was measured using the reflection-type THz-TDS system. As shown in Fig. 2C, the roughness affected the reflectance spectra, and the reflectance decreased with increasing roughness compared with that of a smooth plane. In this study, the term “random assumption (ASMP)” is used to describe phantoms with a surface microstructure, as opposed to periodic or regular patterns. A significant decrease in reflectance was observed for the random ASMP at higher frequencies. This tendency of reflectance decrease can be represented at the *a* position by calculation using models with random roughness (Gaussian distribution) as mentioned above. However, we found that the calculation results differed from the measurement results at the *b* position. The reflectance values at the *a* and *b* positions were similar at 0.3 THz. Thus, we considered that the agreement between the calculation and measurement results depended on not only the roughness but also the surface shape.

### Reflectance properties of reference materials

**Fig. 3:**
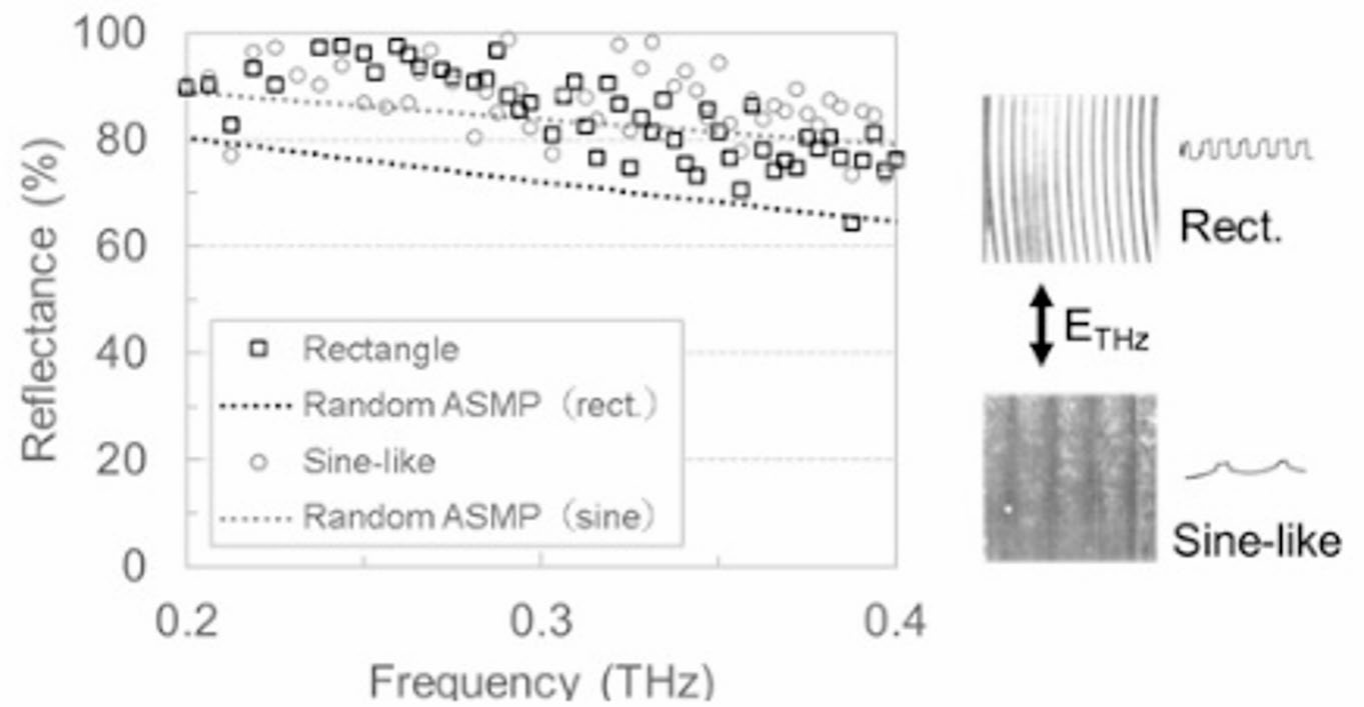
Reflectance spectra of surface-roughness-standard pieces with rectangular or sine-like shape. Calculation results with assumption of random roughness were also indicated by black or grey dotted lines for their roughness.

To discuss the shape dependence in the reflectance, reflectance spectra of two surface-roughness-standard pieces were measured using the same reflection-type THz-TDS system. One piece has a lattice pattern, peaks and valleys with a depth of 0.06 mm and a period of 2 mm [sine-like shape, roughness (RMS) ~ 0.014 mm]. Another piece has peaks and valleys with a depth of 0.07 mm and a period of 0.6 mm [rectangular shape, roughness (RMS) ~ 0.026 mm]. When we measured the roughness of standard pieces, the polarization direction of a THz pulse was parallel to the lattice. As shown in Fig. 3, the rectangular piece had reflectance similar to that of the sine-like shape, even though the roughness was larger.

Furthermore, the calculation results differed from the measurement results for the rectangular shape. We considered that a reason for the difference was the high ratio of specular elements to rough elements. Thus, surface shapes affect reflectance, and a random pattern with fewer specular elements causes significant changes in reflectance.

**Fig. 4:**
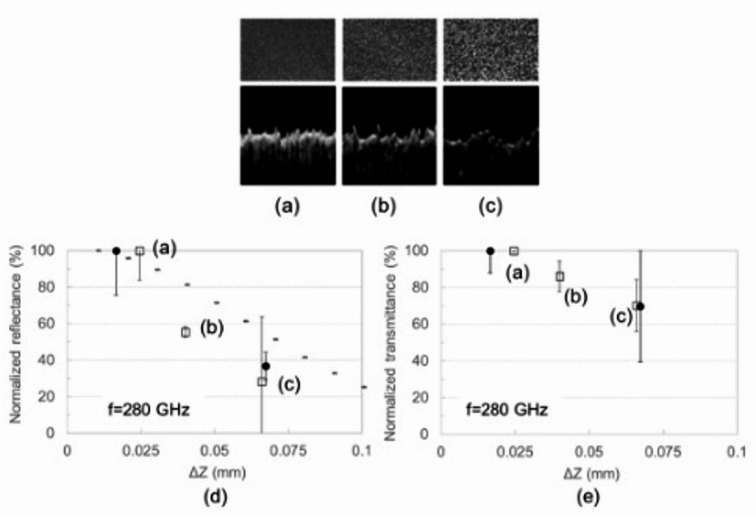
Photographs and OCT images of sandpaper with roughnesses (RMS) of approximately (**a**) 0.025 mm, (**b**) 0.040 mm, and (**c**) 0.066 mm. The relationships of normalized (**d**) reflectance and (**e**) transmittance with surface roughness of sandpaper (squares) and skin-equivalent phantoms (circles) at 0.28 THz are shown. Calculation results with the assumption of random roughness are also indicated by a grey dashed line in the left graph.

We analyzed the relationship between reflectance and transmittance using sandpaper with random surface patterns, as shown in Figs. 4(a)–4(c). Here, three types of sandpaper with roughness (RMS) of approximately 0.025, 0.040, or 0.066 mm were used. The relationships of surface roughness with reflectance and transmittance normalized at the smallest roughness at 0.28 THz are plotted in Figs. 4(d) and 4(e), respectively. Error bars indicate the standard deviation of four measurements. Results show that both reflectance and transmittance decreased with roughness. The error bars shown in Fig. 4(e) are most likely related to roughness deviation.

The reflectance and transmittance of skin-equivalent phantoms were also measured. The skin-equivalent phantoms had surface roughnesses (RMS) of approximately 0.017 and 0.067 mm. The relationships of surface roughness with reflectance and transmittance normalized at the smallest roughness are plotted in Figs. 4(d) and 4(e), respectively. We verified that the relative transmittance tendencies agreed well with those of the sandpaper, regardless of the material difference, even though the actual transmittance values differed.

### Phantom preparation

**Fig. 5:**
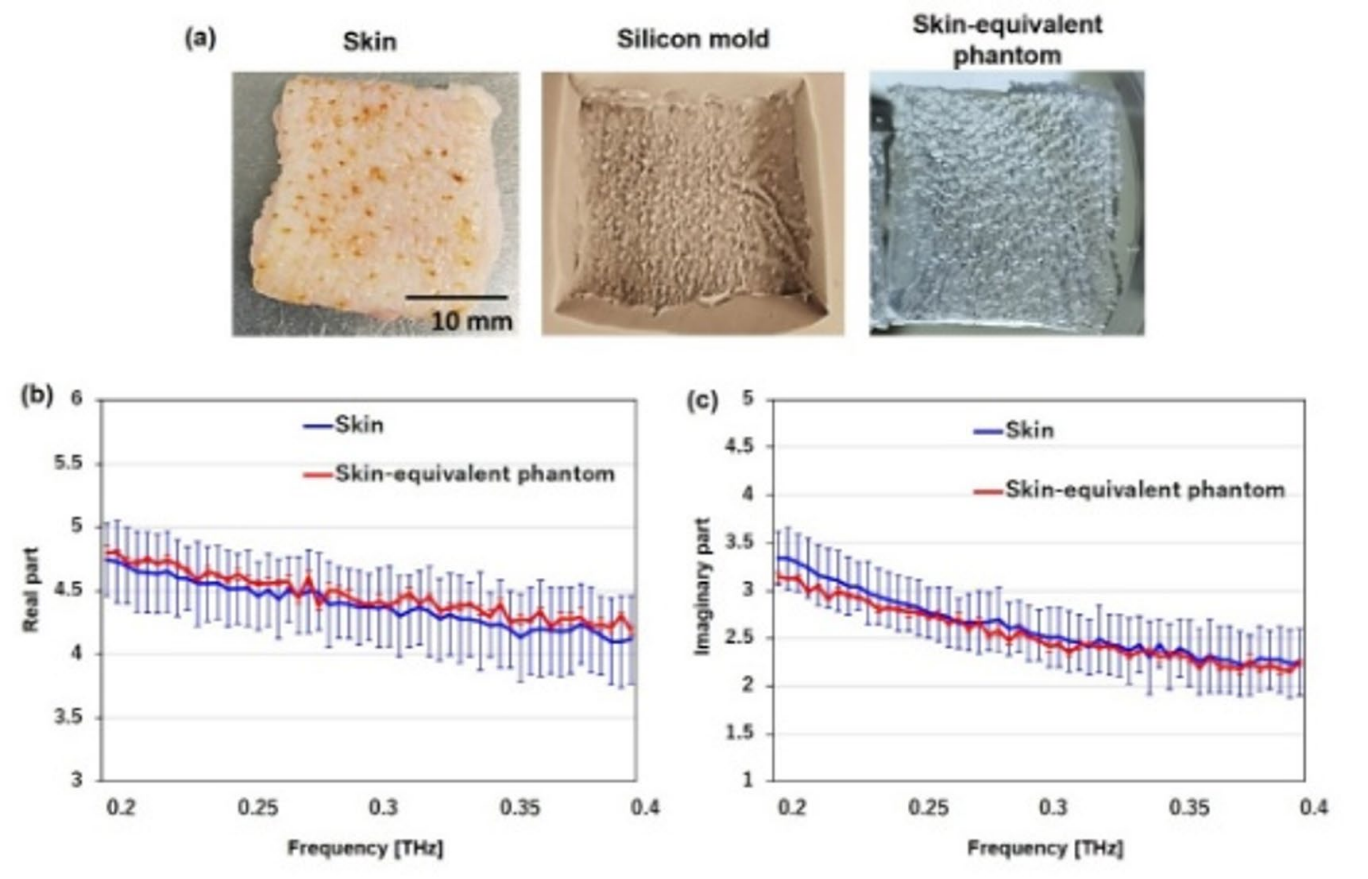
(**a**) Photographs of human skin, a silicon mold of the skin, and a skin-equivalent phantom fabricated using the mold. (**b**) Real and (**c**) imaginary parts of the complex relative permittivity of human skin (blue) and the skin-equivalent phantom (red) measured at frequencies from 0.2 THz to 0.4 THz. Data represent the mean ± standard deviation for skin (*n* = 10) and skin-equivalent phantom (*n* = 5).

**Fig. 6:**
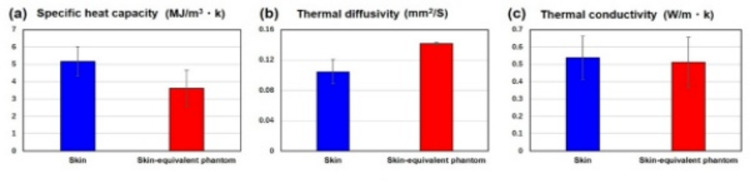
Thermal properties of porcine skin and the skin-equivalent phantom. (**a**) Specific heat capacity (MJ/m³·K), (**b**) thermal diffusivity (mm²/s), and (**c**) thermal conductivity (W/m·K). Error bars indicate standard deviations based on multiple measurements (*n *= 8).

On the basis of the results of the reflectance and transmittance measurements, a skin-equivalent phantom was prepared for evaluating temperature increases under 265 GHz exposure. As shown in Fig. 5(a), a silicon mold was fabricated to replicate the surface texture of porcine skin. This mold was created by casting the skin surface, allowing the phantom to faithfully reproduce the microtopography and roughness of the skin (RMS ≈ 0.10 mm). By incorporating this detailed surface structure, we obtained a phantom that better simulates the optical and thermal interactions of real skin with THz waves. Next, the complex relative permittivity of the skin-equivalent phantom was adjusted to match that of the skin. Figures 5(b) and 5(c) show the real and imaginary parts of the complex relative permittivity of the skin and imitation phantom, respectively. Mean values with standard deviations (1σ) calculated from ten skin samples are presented. The maximum measurement S.D. were 9.0% for the real part and 15.4% for the imaginary part. A skin-equivalent phantom was prepared using 69.5% saline solution, 30% ethanol, and 0.5% agar. The complex relative permittivities, both real and imaginary, of the skin (blue) and the phantom (red) were in close agreement, with the phantom values falling within the error margins of the skin throughout the 0.2–0.4 THz frequency range. In addition, the thermal responses of the skin and phantom were examined and compared. The thermophysical properties of porcine skin and the fabricated skin-equivalent phantom are presented in Fig. 6. Although minor discrepancies are apparent in specific heat capacity [Fig. 6(a)], thermal diffusivity [Fig. 6(b)], and thermal conductivity [Fig. 6(c)], the absolute differences remain within a limited range. Given the relatively small magnitude of the vertical axes and the reported variability in biological tissue properties^21,22^, these deviations are unlikely to significantly affect heat transfer behavior under realistic exposure conditions. Accordingly, the phantom can be regarded as thermophysically analogous to biological skin and is thus suitable for simulating thermal responses in electromagnetic exposure assessments.

### Temperature increase measurements under 265 GHz exposure

THz waves are strongly absorbed by liquid water, which restricts their penetration in biological tissue to a few hundred micrometers. As a result, energy absorption leads to temperature rises primarily near the tissue surface. Conventional sensors, such as fiber optic thermometers, have a spatial resolution of approximately 1 mm, limiting their ability to capture these fine-scale temperature changes^23^. To overcome this, we employed fluorescent thermoprobes embedded within the phantom. Fluorescence was detected using a confocal laser microscope, allowing temperature distributions to be mapped with 2 μm transverse (xy) and 20 μm axial (z) resolution and a temperature resolution of 0.04 °C^19^. To measure the temperature increase of the skin-equivalent phantom induced by THz waves, the phantom containing fluorescent probes was irradiated with 265 GHz waves generated by a gyrotron^15^. The temperature increases were measured at three power densities: 100 mW/cm², 150 mW/cm², and up to a maximum of 200 mW/cm². To determine the power densities, we referenced previous studies showing thermal damage to biological tissues under 162 GHz exposure^24^. In our experimental setup, a wire grid was used to adjust the power density of the irradiated waves^16^. The local THz power density at the sample position was measured using a setup based on the waveguide probe setup described in the *Measurement system* section of Methods.

**Fig. 7:**
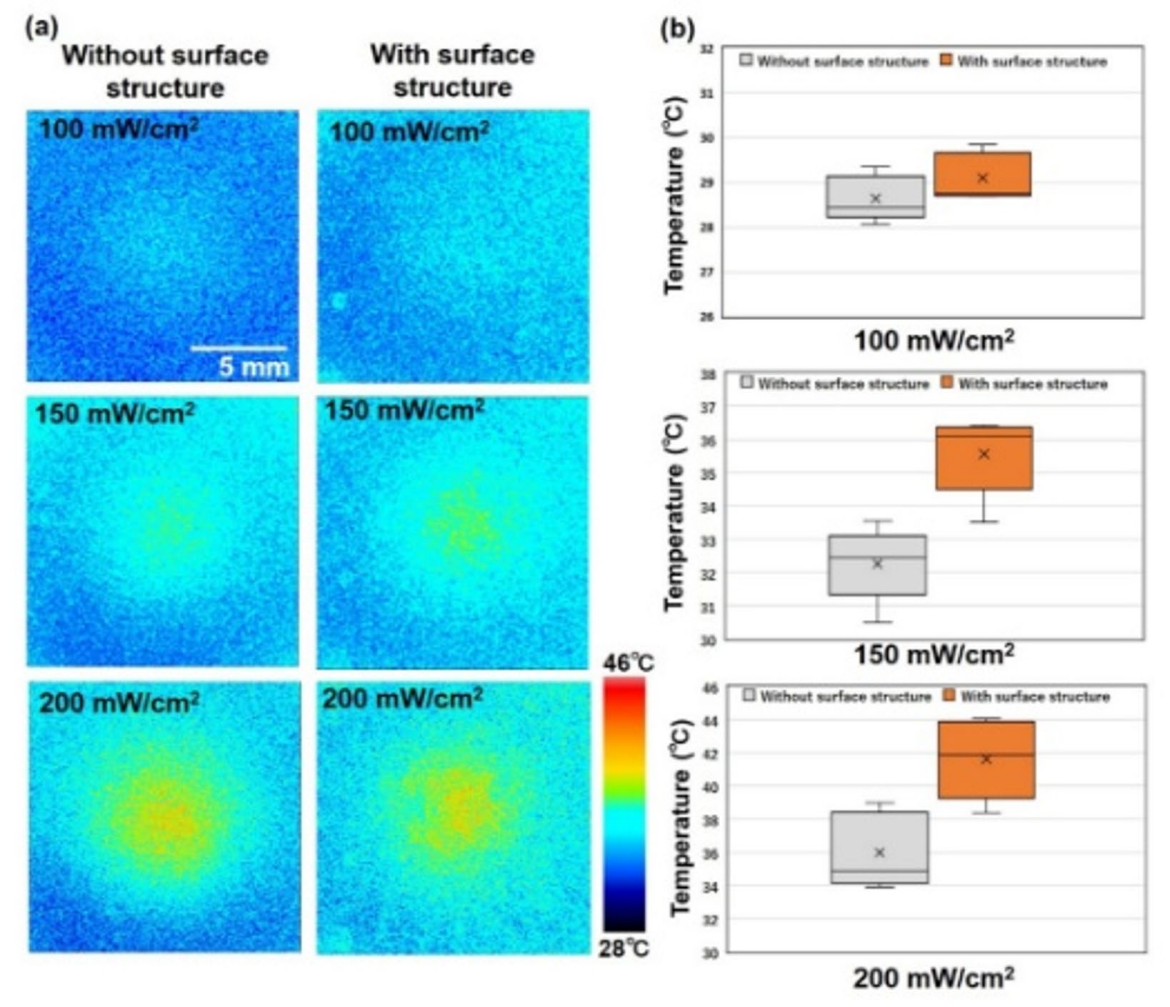
Surface temperature distributions of skin-equivalent phantoms without and with skin surface structures under 265 GHz wave exposure. (**a**) Temperature maps obtained using confocal laser microscopy. (**b**) Box plots showing average surface temperatures of the phantoms under each condition (*n* = 5). The x mark in each box represents the median value.

Figure 7(a) shows the surface temperature distributions of the skin-equivalent phantoms without and with skin surface structures under exposure to 265 GHz waves at different power densities. After 10 min of exposure, when the phantom had reached thermal equilibrium, temperature measurements were performed. Figure 7(b) shows boxplots of the five samples average temperatures in the regions of interest for each condition. At a power density of 100 mW/cm², no significant temperature difference was observed between the phantoms with and without surface structures. In both cases, the average temperatures remained around 29–30 °C, which is comparable to the ambient room temperature (29 ± 2 °C), indicating no measurable temperature increase. Note that the experimental environment was maintained at a temperature close to that of the human skin surface, approximately 30 °C, to ensure physiological relevance^25^. On the other hand, at higher power densities of 150 and 200 mW/cm², the phantom with skin surface structures exhibited higher surface temperatures than the phantom without structures. Specifically, at 150 mW/cm², the average surface temperature of the phantom with surface structures was approximately 35.5 °C, whereas that without surface structures was around 32.5 °C. At 200 mW/cm², the temperatures of the phantoms with and without surface structures were approximately 42 and 36 °C, respectively. These findings suggest that the presence of surface structures on the phantom enhances localized temperature increases under THz wave exposure.

**Fig. 8:**
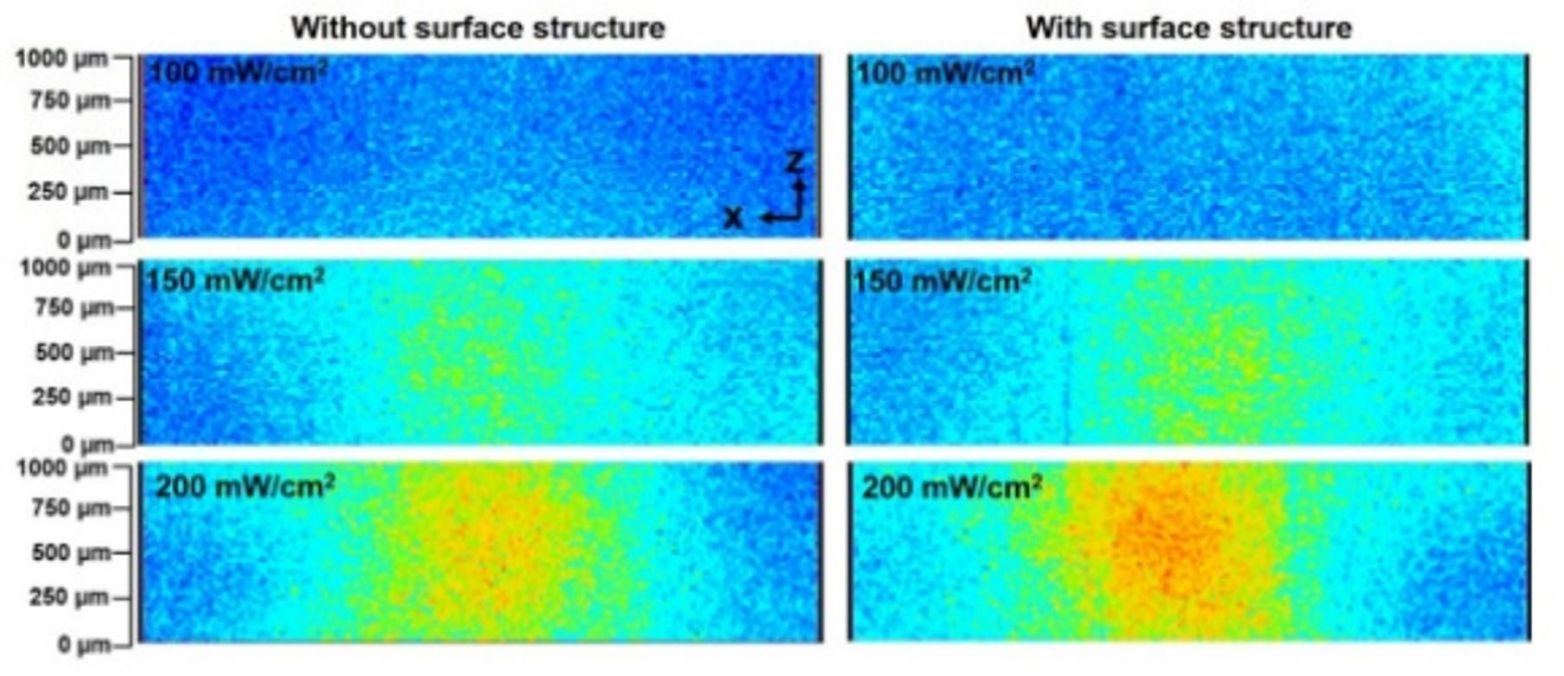
Cross-sectional temperature distributions inside the skin-equivalent phantom under 265 GHz wave exposure at power densities of 100, 150, and 200 mW/cm². For each condition, the phantom without surface structure (left) and with surface structure (right) are shown.

Figure 8 shows cross-sectional temperature distributions inside the skin-equivalent phantom at power densities of 100, 150, and 200 mW/cm². For each condition, images from phantoms without (left) and with (right) surface structures are presented. These cross-sectional images correspond to the central plane of a stack acquired from the exposed surface to a depth of 1 mm, with 20 μm step intervals. Although the penetration depth of 0.3 THz waves in biological tissue is generally limited to several hundred micrometers, the observed temperature elevations extended up to 1 mm into the phantom. This suggests that the heat generated at the surface diffused into the deeper regions of the phantom through thermal diffusion. These results indicate that, for the accurate assessment of temperature increase induced by THz wave exposure, it is essential to evaluate not only surface temperature but also internal temperature distributions.

## Discussion

We found that surface roughness affected the reflectance of the porcine skin, skin-equivalent phantom, and reference materials such as standard pieces and sandpapers. On the basis of the reflectance measurement results of reference materials, we verified that the effect of surface roughness differed depending on the roughness and area of specular elements, and became maximum when the roughness was random. In the porcine skin measurements, fine-grained skin (with uniform roughness) had a reflectance spectrum similar to that of the random roughness calculation model, whereas the reflectance spectrum of skin having deep grooves significantly differed from that of the random roughness calculation model. Because porcine skin reflectance decreased by a few percent compared with a smooth plane, the influence of roughness should also be considered in human skin at frequencies above roughly 0.3 THz. The observed variations in reflectance mainly result from a surface roughness on dielectrics. While our modeling supports the presence of interference between adjacent waves phase-shifted on dielectrics with surface roughness, contributions from an effective medium are also considered. In the same manner, surface roughness also affected the transmittance of the skin-equivalent phantom. By calculating the superposition, when the skin-equivalent phantom has a random roughness of 0.05 mm, we can calculate the reflectance and transmittance of the skin at 1 THz as less than 2 and 50% of the original value, respectively. The transmittance is directly related to THz energy absorbed into the human skin. If we use the plane phantom for exposure measurements, maximal transmittance, and thus the worst exposure condition, could be achieved; the THz energy absorbed into the human skin would be overpredicted. Although it can be conservatively discussed, we consider that investigating human skin roughness and developing skin-equivalent phantoms with human skin roughness are also required to clarify certain THz absorption energy in the human skin at frequencies above 0.3 THz.

Notably, phantoms with surface structures displayed higher temperature elevations compared to flat phantoms once thermal equilibrium was reached. This suggests that factors other than optical properties, such as enhanced water evaporation and localized heating, contribute to the observed thermal behavior. To investigate the mechanism behind this temperature increase, we quantified transepidermal water loss (TEWL) on the phantom surfaces using a Tewameter TM HEX. The results, presented as box plots in Supplementary Fig. S2, showed that the average TEWL value for the phantom with surface structures was 39.52 g/m²/h, which was higher than that of the flat phantom at 35.5 g/m²/h. This indicates that surface structures promote water evaporation, resulting in slight reductions in surface water content and temperature. As THz waves are strongly absorbed by water, the slight reduction in surface water temperature may enhance energy penetration into subsurface regions, resulting in increased internal heating. This mechanism provides a plausible explanation for the unexpectedly higher temperatures observed in the structured phantom, despite the expectation that surface roughness would reduce THz energy deposition owing to increased scattering. To further support the validity of our structured phantom, it is noteworthy that the human skin exhibits microstructural features such as furrows and ridges, which are associated with localized variations in hydration and TEWL. Previous studies have shown that TEWL and stratum corneum water content vary significantly across different facial and body sites^26,27^, and these differences are affected by surface texture and microanatomy^28^. This aligns with our finding that phantoms with surface structures showed elevated TEWLs and greater internal temperature increases, indicating that these structured phantoms more closely mimic the physiological features of human skin regarding THz thermal interactions.

These results suggest that the dominant factors affecting THz–tissue interactions are related to the exposure duration. Reflectance and transmittance, altered by surface roughness, significantly affect the initial THz energy absorption in short-term exposure. In long-term exposure, the gradual decrease in surface water content promotes deeper penetration and absorption of THz waves, leading to temperature increases governed more by subsurface interactions. Although the instantaneous reflectance differences with water content appear minor (Fig. 1b), even modest evaporation of surface water during prolonged exposure reduces THz attenuation as well as the reflectance in the superficial layer. This allows more THz energy to reach subsurface regions, gradually enhancing internal heating over time. Therefore, the cumulative effect of water loss meaningfully contributes to subsurface temperature increases, which may not be captured by surface reflectance measurements including individual deviation of porcine skin. Accordingly, phantom design and evaluation methods should be adapted to the exposure scenario: optical characterization is more relevant for short-term, localized exposures, whereas combined optical and thermal properties are necessary for long-term irradiation. In this study, we focused on a fixed frequency of 265 GHz because it corresponds to the operational frequency of our exposure system and lies within a region of interest for safety standard discussions. For broadband THz exposure, higher-frequency components are expected to experience stronger absorption due to the frequency-dependent increase of water’s absorption coefficient. This would lead to more pronounced surface heating and steeper thermal gradients near the surface, while deeper regions remain less affected, compared with a single representative frequency. Considering such frequency-dependent effects will be important for extending our findings to practical, broadband THz irradiation scenarios. Nevertheless, our primary aim here was to clarify exposure characteristics at a single representative frequency under practical conditions. Future work will extend this approach to broadband measurements to examine how the characterization evolves across the THz spectrum. A clear understanding of these distinctions will be essential for establishing safety guidelines and standardized evaluation protocols in the THz frequency range.

## Conclusion

In this study, we investigated the reflectance and transmittance of skin-equivalent phantoms with surface roughness in the frequency range of 0.2–0.4 THz. The results showed that surface roughness reduced both reflectance and transmittance. These findings indicate that reproducing the surface microstructure of the human skin is important for accurately estimating THz absorption in real skin at a frequency of around 0.3 THz. We also developed a phantom embedded with fluorescent thermoprobes to evaluate temperature increases caused by THz wave exposure. Contrary to expectations based on optical properties, phantoms with surface structures exhibited higher temperature increases than those with flat surfaces. This suggests that surface geometry can significantly affect thermal behavior and should be considered in exposure assessments. These results demonstrate the need to integrate both optical and thermal evaluations when assessing the biological effects of THz radiation. Further development of skin phantoms that replicate realistic surface conditions will help improve the accuracy of such evaluations.

## Supplementary Information

Below is the link to the electronic supplementary material.

Supplementary Material 1

## Data Availability

The data supporting the findings of this study are available from the corresponding author upon reasonable request.
