# Supplementary material for: Investigation of reflectance, transmittance, and thermal response of skin and surface-textured skin-equivalent phantoms in the terahertz frequency range around 0.3 THz
Source: Sci Rep. 2025 Dec 22;15:45042. doi: 10.1038/s41598-025-28863-0 (PMC12748668; doi:10.1038/s41598-025-28863-0)
Supplement: Supplementary file 1 — Supplementary Material 1 [file 41598_2025_28863_MOESM1_ESM.docx]

**Title**

**Investigation of Reflectance, Transmittance, and Thermal Response of Skin and Surface-textured Skin-equivalent Phantoms in the Terahertz Frequency Range Around 0.3 THz**

**Authors**

Shota Yamazaki^1^*, Masafumi Fukunari^1,2^, Yoshinori Tatematsu^1,2^, Yujiro Kushiyama^1^, Tomoaki Nagaoka^1^, Maya Mizuno^１^

**Affiliations**

^1^National Institute of Information and Communications Technology, Koganei, Tokyo 184-8795, Japan

^2^University of Fukui, Bunkyo, Fukui 910-8507, Japan

*Corresponding author: correspondence should be addressed to syamazaki@nict.go.jp

**Supplementary Figures**


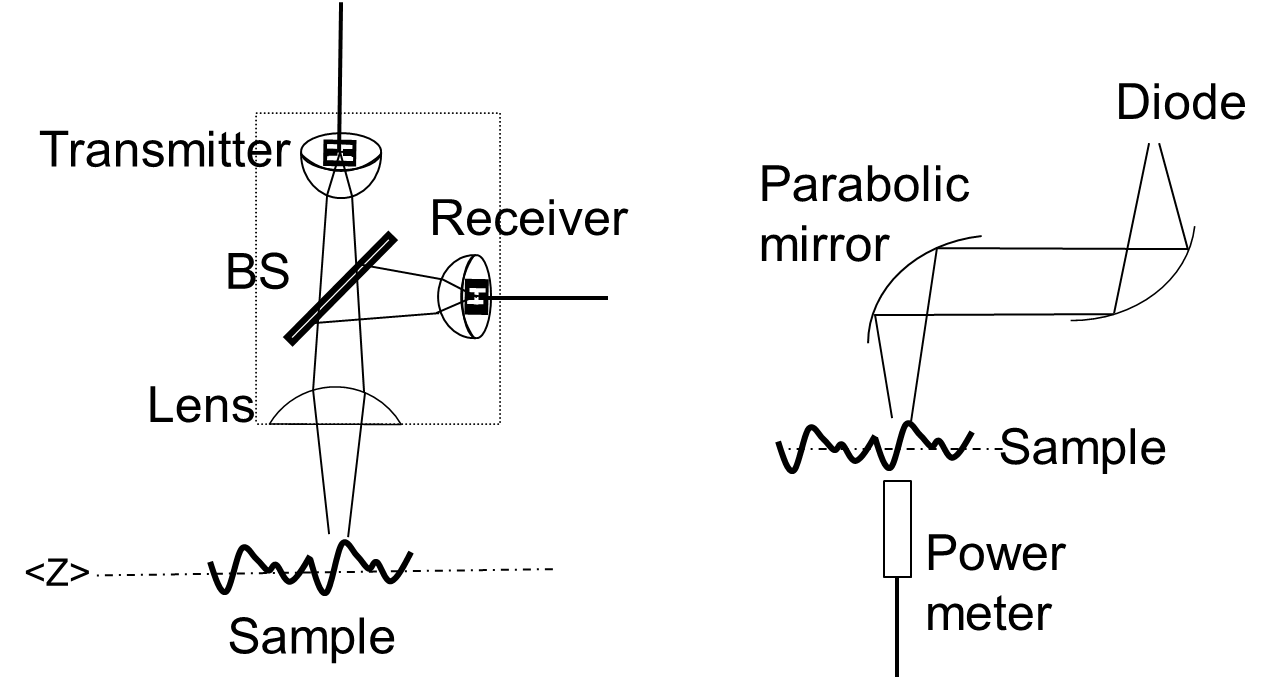


Supplementary Figure S1. Schematics of optical configurations for reflectance (left) and transmittance (right) measurements.


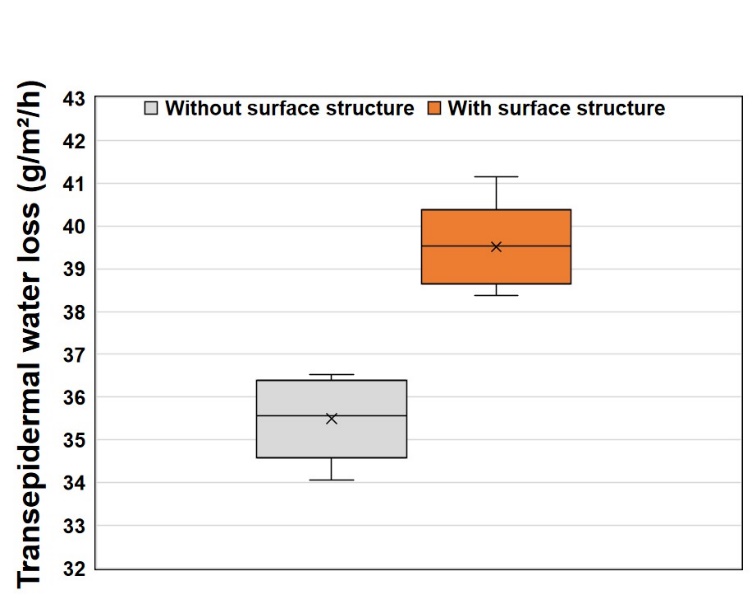


Supplementary Figure S2. Transepidermal water loss of skin-equivalent phantom with and without skin surface structures.
